# Stafne bone defects radiographic features in panoramic radiographs: Assessment of 91 cases

**DOI:** 10.4317/medoral.22592

**Published:** 2018-12-24

**Authors:** Miki Hisatomi, Luciana Munhoz, Junichi Asaumi, Emiko-Saito Arita

**Affiliations:** 1 DDS, PhD. Department of Dentomaxillofacial Radiology and Oral Diagnosis, Okayama University Hospital, 2-5-1 Shikata-cho, Zip Code 700-8558 Okayama, Japan; 2 DDS, MS. Department of Stomatology, School of Dentistry, University of São Paulo, 2227 Lineu Prestes Avenue. Zip Code 05508-999 São Paulo, SP, Brazil; 3 DDS, MDSc. Department of Oral and Maxillofacial Radiology, Okayama University Graduate School of Medicine, Dentistry and Pharmaceutical Sciences, 2-5-1 Shikata-cho, Zip Code 700-8525 Okayama, Japan; 4 DDSm, PhD. Department of Stomatology, School of Dentistry, University of São Paulo, 2227 Lineu Prestes Avenue. Zip Code 05508-999 São Paulo, SP, Brazil

## Abstract

**Background:**

To evaluate 91 cases of Stafne bone defect (SBD) in panoramic radiographs (PR) to determine the prevalence of different SBD variants, considering age, gender, and side. Additionally, to assess the most frequent imaging features of SBD.

**Material and Methods:**

Participant data were collected from 91 SBD cases with PR imaging. First, SBDs were classified according to their location, as anterior, posterior, or ramus variant. SBD imaging features were classified according to radiographic imaging findings, assessing margins, degree of internal radiolucency, shape, topographic relationship between the defect and mandibular border, location of the defect according to mandibular teeth, and locularity. The topographic relationship between the SBD and the mandibular canal was described for the inferior variant only. Mean sizes were also described.

**Results:**

A total of 92 SBD cases were evaluated from 91 radiographs. One case presented multiple defects. Mean patient age was 60.80 years. Men were more affected than women. The most frequent SBD variant was the posterior variant, and the least frequent was the ramus variant. The most observed radiographic features were thick sclerotic bone margin in the entire contour of the defect, partially radiolucent internal content, oval shape, continuity with mandible base without discontinuity of mandible border, third molar region location, and unilocular shape. With the posterior variant only, the most common topographic relationship between the defect and the upper wall of the mandibular canal was the defect located below the upper wall and continuous with the inferior wall of the mandibular canal.

**Conclusions:**

The knowledge of common SBD radiographic imaging features in PR can help dental practitioners with the differential diagnosis of SBD.

** Key words:**Panoramic radiograph, mandible, bone cysts, salivary glands, Stafne bone defect.

## Introduction

Stafne bone defect (SBD) is a radiolucent depression or defect in the mandible first described in 1942 (1). Since then, multiple terms have been used to describe this depression, such as “Stafne bone cyst”, “Stafne bone cavity”, “latent bone cyst”, “aberrant salivary gland defect”, “developmental bone defect of the mandible”, “idiopathic bone cavity”, or “cortical mandibular depression” (2). SBD is defined as a bone cavity or pseudocyst filled mainly by salivary gland tissue(3,4); however, muscles, lymphoid tissue, blood vessels, fat, and/or connective tissue may also be found (3,5).

The etiology of SBD is unclear (6,7). Many theories exist about the origin of the depression, including from a hypertrophic lobe of a salivary gland (7), the result of an erosion from vascular compression (4,5), or due to incomplete Meckel cartilage calcification during ossification (5,8). The diagnosis of SBD is often incidental (9,10) due to the asymptomatic nature of the defect (2,7,9); in very rare cases, SBD can be palpated (11).

In conventional radiographs, SBD typically resembles a unilocular cystic lesion (7) with well-defined borders, although a multilocular appearance and ill-defined borders have been reported (4). Radiolucency shape may be round or oval (7,10). The same features are seen in computed tomography (CT), with the advantage of possible investigations on cortical wall perforations or expansion (12). CT scans also show SBD limits with relatively high signal intensities compared to neighboring muscles, (13) as well as adjacent structures related with the defect. Magnetic resonance imaging (MRI) can confirm the presence of salivary tissue on T2 and T1-weighted multiplanar imaging (14) or show other soft tissue filling the depression (13).

SBD presents as four variants: lingual posterior, lingual anterior, lingual ramus (15), and buccal ramus depression (16). In panoramic radiographs, SBD variants are often described according to their location in the mandible: posterior, anterior (17), or ramus (8). The most frequently observed variant is the lingual posterior, typically located near the angle of the mandible and under the inferior alveolar canal on panoramic radiographs (6,7,10). The presence of multiple simultaneous defect variants in the same patient have been reported, but this is rarely seen (18).

The radiographic prevalence of SBD on radiographic studies is low, with less than 0.5% to the posterior lingual variant in the total population (3,7), and differences in SBD prevalence ranges widely between studies (17). To our knowledge, few studies examining the prevalence of the distinct variants in a large number of SBD cases have been previously published; none have explored common imaging features and mean sizes of SBDs in panoramic radiographs. This study is relevant because it elucidates essential imaging characteristics of SBD in panoramic radiographs, which are important radiographic examinations in routine dentistry practice. Panoramic radiography is the primary tool to detect SBD due to its broad use in routine practice, often requested at the beginning of dental treatments, and because most patients lack symptoms.

Thus, the objective of this study was to evaluate 91 SBD cases, identified in panoramic radiographs, to determine the overall prevalence of the different SBD variants, as well as SBD prevalence by patient age, gender, and side. Additionally, the study assessed the most common imaging features of SBD, such as lesion margin characteristics (thickness, presence or absence of bone sclerosis), the degree of radiolucency of the internal structure (radiolucent, partially radiolucent), defect shape (round defect, oval-shaped defect), the topographic relationship between the depression and the mandible border, detailed localization of the depression (according to mandibular teeth), locularity, relationship between the mandibular canal and the bone defect (in the posterior variant), and the sizes of the distinct variants (mean, maximum, and minimum).

## Material and Methods

-Inclusion and exclusion criteria

91 cases were selected in which SBD had been previously detected and confirmed in panoramic radiographs. Radiographs with technical failures were not included in the sample, nor were examinations with lesions or alterations in the area of interest or adjacent areas. Participants’ data, such as age and gender, were collected. Ethics Committee approval was obtained from the university (number: CAAE 82037317.9.0000.0075)

-Radiographic methods and panoramic radiograph evaluations

The panoramic radiographs were performed by the same device, and images were processed and measured using the same software (ImageJ, National Institutes of Health, Bethesda, MD, USA).

First, SBDs were classified by three variants according to their location, as described:

• Anterior variant: The defect was in the sublingual gland area, in the anterior mandible region, or in mandible body.

• Posterior variant: The defect was in the submandibular gland area, which is in the posterior region of the mandible.

• Ramus variant: The defect was in the parotid gland area, which is in the ramus of the mandible.

**Figure 1:**
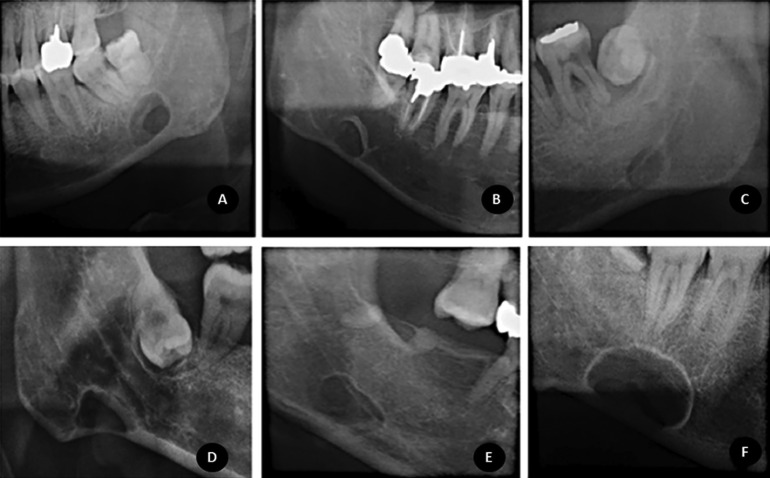
Examples of bone margin and locularity classifications, from left to right: Figure 1A demonstrates a multilocular defect with total thin sclerosis; 1B demonstrates a unilocular defect with thick sclerosis; 1C demonstrates a unilocular defect without sclerosis at bone margins. Examples of the topographic relationship between the mandibular canal and the defect: Figure 1D shows a defect below the mandibular canal; Figure 1E shows a defect overlapping the mandibular canal inferior wall and below the mandibular canal upper wall; Figure 1F exhibits a defect that overlaps the upper and inferior walls of the mandibular canal.

**Figure 2:**
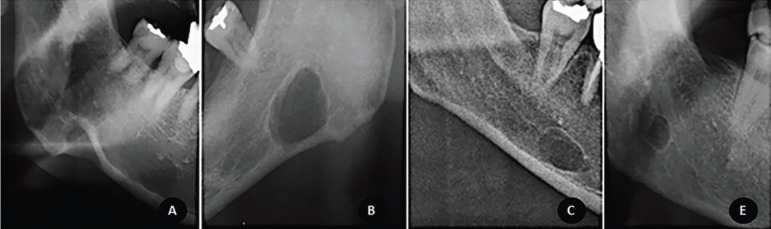
Examples of topographic relationships to the mandible border. From left to right: Figure 2A represents a defect continuous to the mandibular border with clear discontinuity of mandible cortex, Figure 2B represents a defect continuous to the mandibular border without discontinuity of mandible cortex, Figure 2C exhibits a defect continuous to the mandible base; Figure 2D shows a defect distant from the mandible base.

Next, SBD imaging features were analyzed by three observers, and the radiographic characteristics were classified as follows:

1) Margins: defects in bone margins were defined, according to the presence of any sclerosis, as thin sclerosis, thick sclerosis, or no without sclerosis. When sclerosis was present, it was also classified as partial (when sclerosis was not on the entire contour of the defect) or total (when sclerosis was present on the entire contour of the defect). Figure 1 (A,B,C) demonstrates examples of bone margin classification.

2) Internal radiolucency degree: internal radiolucency was defined as partially radiolucent (when the presence of any bone trabeculae within the defect was detected) or totally radiolucent (when no bone trabeculae were observed within the defect).

3) Shape: classified as oval or round.

4) Topographic relationship between the defect and the mandibular border: determined as defect continuity to mandible base (with or without visible discontinuity of mandible cortical cortex), defect contiguity with mandible base, and/or absence of contiguity/continuity with mandibular border (the defect does not touch the mandible base or mandible cortex). Examples of topographic relationship to the mandibular border are depicted in Figure 2.

5) Localization of the defect: according to its proximity to mandibular teeth or its region, except for the ramus variant.

6) Locularity: classified as unilocular or multilocular. Figure 1 also demonstrates examples of defect locularity.

7) For the posterior variant only, the topographic relationship between the mandibular canal and the defect were detailed as: a) below the mandibular canal inferior wall (the defect does not touch the inferior mandibular canal wall); b) below the mandibular canal upper wall and continuous with the mandibular canal inferior wall; c) below the mandibular canal upper wall and contiguous with the mandibular canal inferior wall; d) below the mandibular canal upper wall and overlapping the mandibular canal inferior wall (when the wall can be observed within the defect); e) overlapping the mandibular canal upper and inferior wall; f) continuous with the mandibular canal upper wall; g) contiguous with the mandibular canal upper wall; h) above the mandibular canal (the defect does not touch the mandibular canal superior wall). These classification examples are shown in Figure 1 (D,E,F).

Finally, the side of the defect (right, left, or both) was recorded along with mean sizes of the distinct SBD variants.

-Statistical analysis

Descriptive statistics

Mean, minimum, and maximum ages of the participants were detailed according to gender. The number and percentage of distribution of each SBD variant were described, as well as the percentages of cases exhibiting the aforementioned imaging features, as evaluated in panoramic examinations, along with each variant’s maximum, minimum, and mean size (stated in millimeters). Statistical evaluation was performed using IBM SPSS Statistics 24 (SPSS, Inc., Chicago, IL, USA).

## Results

A total of 91 panoramic radiographs with the presence of SBD were evaluated; the total number of defects evaluated was 92 (90 radiographs had a single defect, and 1 had multiple defects).

-Gender and age characteristics of participants affected by SBD

The mean age of the patients affected by the defect was 60.8 years old. The minimum age was 28, and the maximum age was 83 years old. Detailed data about participants’ ages are depicted in  Table 1. More males were affected than females; the more common side was the right side, as shown in  Table 1.

**Table 1:**
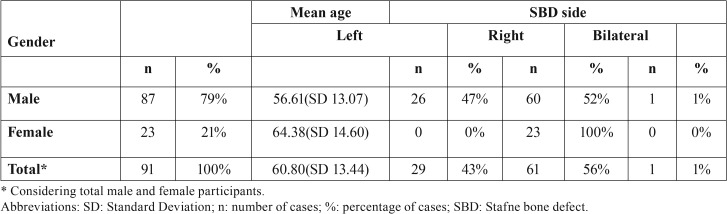
Number and percentage of male and female participants, mean age, and side affected by SBD.

-Number and percentages of each SBD variant

**Figure 3:**
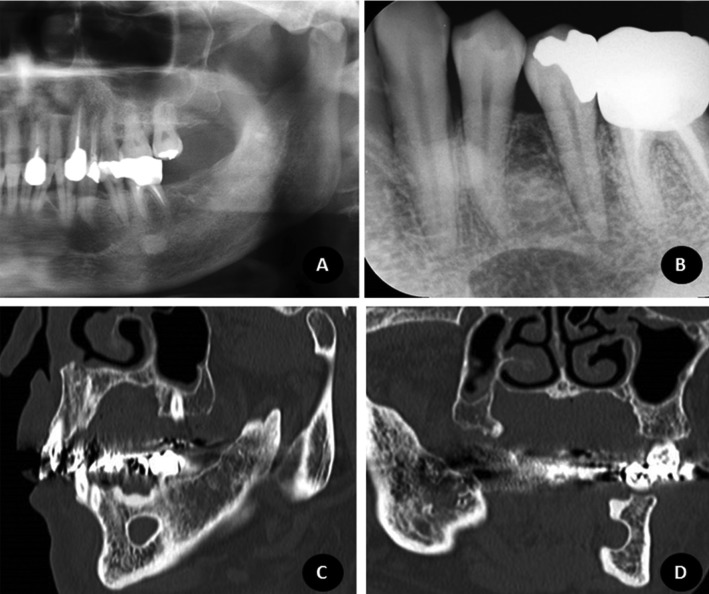
An example of an SBD case in the premolar area from the sample. Figure 3A shows the panoramic radiograph detail of the case; Figure 3B the periapical radiograph; Figure 3D and 3E demonstrate sagittal and coronal slices in multislice CT.

The most frequent SBD variant was the posterior variant, and the least frequent was the ramus variant, as shown in  Table 2,  Table 2 continue. Two defects were found between premolars. In Figure 3 (A,B), an SBD in the premolar area is demonstrated in a panoramic radiograph detail and periapical radiograph. Figure 3 (C,D) shows further examinations of this case in multi-slice CT.

**Table 2:**
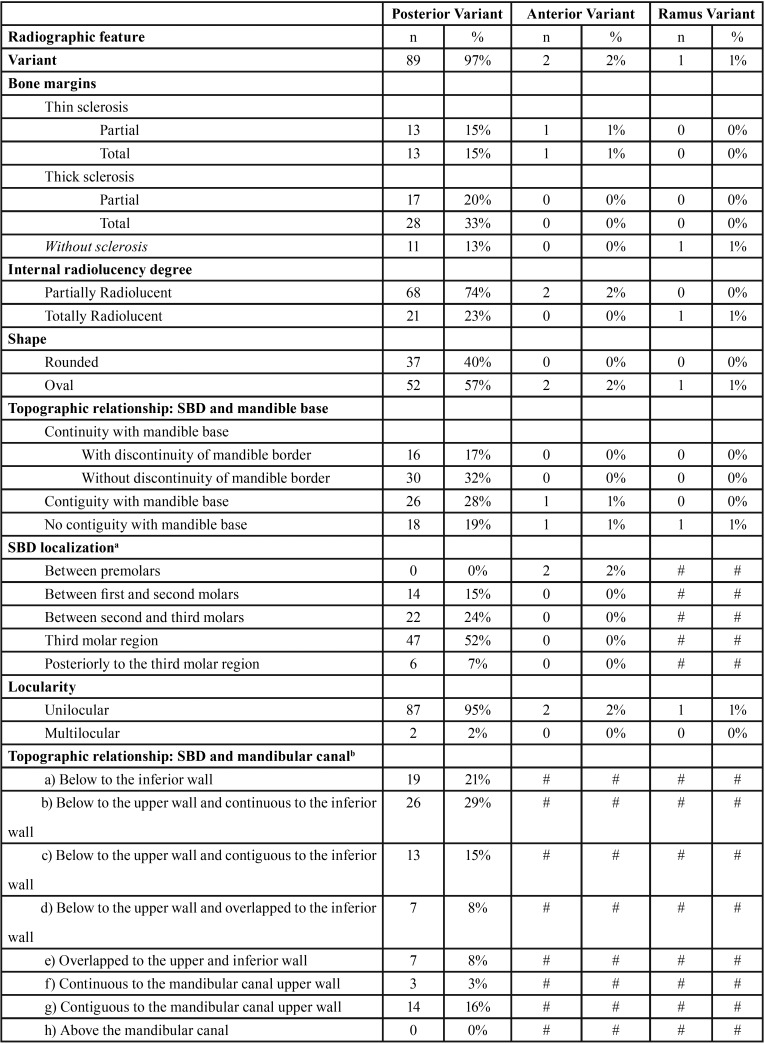
Number of cases and percentages of each Stafne bone defect variant and number of cases and percentages.

**Table 2 continue:**
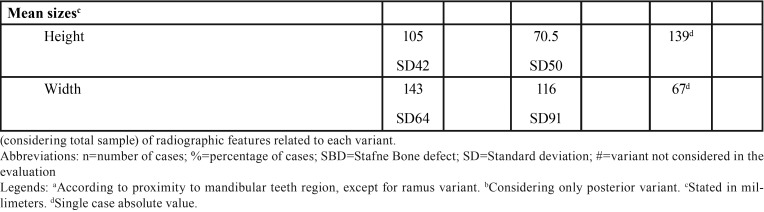
Number of cases and percentages of each Stafne bone defect variant and number of cases and percentages.

-Radiographic features

Radiographic features and mean sizes of each variant are described in  Table 2,  Table 2 continue. The most prevalent features were thick sclerotic bone margin in the entire contour of the defect, partially radiolucent internal content, oval shape, continuity with mandible base without discontinuity with the mandible border, third molar region location, and unilocularity. In the posterior variant only, the most observed topographic relationship between the defect and the mandibular canal upper wall was a location below the upper wall and continuous to the inferior wall of the mandibular canal.

## Discussion

In this study, 91 cases of SBDs were evaluated, specifically, three distinct variants in panoramic radiographs. Male participants were more frequently affected than females, and the depression was detected in patients with an age range of 28 to 83 years old (mean age: 60.80 years old). Our age range, mean age, and gender predilection results are similar to previous investigations that show that the depression has a clear predilection for males (6,7) in the fifth or sixth decade, despite the wide age range of affected patients (3,6,7).

The posterior variant was the most prevalent in the present study, in concordance with a previous report that examined a considerable number of SBDs (7). Although the prevalence of the posterior variant in the total population is low, 0.10% to 0.48% in radiographic examinations (17), and 1.3% to 6.06% in cadaver investigation (3,17), this variant is considered typical of SBDs and is often considered as a diagnostic hypothesis in panoramic radiographs (6). The anterior variant is about seven times less common than the posterior variant (14,19), and the ramus variant is quite uncommon (2,7,20), which is in agreement with our results. The presence of simultaneous distinct variants in the same patient is even rarer, but has been described (8,18), and is in concordance with this study’s findings, when only a single case of multiple defects was found.

Despite their prevalence, SBD radiographic features in panoramic radiographs have not been previously described in detail. When we analyzed the SBD borders, we noticed that thick sclerotic margins were more prevalent than thin sclerotic margins, and that the absence of sclerotic margins was rare. Thick sclerotic margins in SBD may resemble cysts (19,21) and have been discussed in many recent case reports (2,19,21,22). The absence of sclerotic borders has already been detailed in other studies (8,11), as have thin sclerotic borders (18). Sclerosis may not be radiographically evident in the entire depression contour if the defect margins are in continuity with the mandible border (20).

The SBD internal radiolucency was much more commonly partially radiolucent than totally radiolucent. This radiographic feature in two-dimensional radiographs, such as panoramic radiographs, may be related to the preservation of the vestibular mandible bone wall, which would be clearly observed in three-dimensional examinations. This characteristic was also noted in prior cases (2,11,22). Furthermore, unilocularity of the SBD was commonly seen in our study. In the literature, multilocular defects are extremely infrequent (23,24) and may raise doubts when considering SBD as a diagnosis.

When SBD shape was evaluated, both types of shape were noted, but the oval-shaped configuration was more common. A predominance of the oval radiolucency shape also has been shown in previous studies (11,12,19).

In our sample, SBD was more often seen in the third molar region. In the literature, most of the reported cases are consistent with our findings and show defects in the third molar region (22,23,25). It is also possible to find depressions in the premolar region (8), between the second and third molars (12), and less commonly, in the angle of the mandible or posterior to the third molar (24).

Continuity with the mandibular border was shown in most of our cases. Previous authors discussed cases with this topographic characteristic (2,11,20,21,23) and note this feature as crucial to differentiating SBD from cystic or neoplastic lesions (6), such as odontogenic tumors, cysts, or inflammatory cysts.

In the posterior variant, our study confirmed that the most common topographic relationship between the mandibular canal and the SBD is where the depression is located inferior to the mandibular canal and mainly inferior to the mandibular canal inferior wall. This was also previously demonstrated in other studies (2,11), although overlap of the inferior and superior mandibular canal wall has also been shown (23).

Mean sizes of posterior and anterior SBD variants were also determined. To our knowledge, this is the first investigation that measured SBD sizes in panoramic radiographs. Previously, SBD volumes were measured by multislice CT (mean 361.7 mm3) (26) and Hounsfield scale values were determined (17).

SBD cases with typical imaging features have an uncomplicated diagnosis (6), especially for the posterior variant (14). Nevertheless, when non-typical defects are encountered, further imaging examination is needed, (7,14,17,27) preferably by radiologic tomographic techniques, such as computed tomography (17,27). Nonetheless, MRI is purported to be the imaging method of choice to confirm a diagnosis of SBD (10,14,28). Salivary gland tissue or other soft tissue in the defect’s interior can be clearly visualized by MRI (10,14,28). Knowledge of the common SBD radiographic features in panoramic radiographs is essential for the professional to refer a patient for appropriate further imaging.

The differential diagnosis for SBD in panoramic radiographs includes benign lesions, such as traumatic bone cysts (29), benign salivary gland tumors, intraosseous hemangiomas, central giant cell lesions, simple bone cysts, fibro-osseous lesions, eosinophilic granulomas, metastatic diseases (7), and ossifying fibromas (30). Lesions such as odontogenic cysts (7) and ameloblastomas may also be considered (29), especially for the anterior SBD variant or if the defect is above the mandibular canal, near to the teeth roots. Patients affected by SBD must be followed with periodic radiographic examinations; SBDs do not show any changes in size and shape over time (7).

In conclusion, our results suggest that the most common variant of SBD is the posterior variant, most often located in the third molar region; the ramus variant is rare. Male patients are more often affected, and the dominant imaging features are thick bone margins, continuity with the mandible border, unilocularity, and partial radiolucence. Knowledge of the common radiographic imaging features of SBD in panoramic radiographs can help dental practitioners with a differential diagnosis of SBD.
